# “It’s Kind of Like Code-Switching”: Black Older Adults’ Experiences with a Voice Assistant for Health Information Seeking

**DOI:** 10.1145/3491102.3501995

**Published:** 2022-04-29

**Authors:** Christina N. Harrington, Radhika Garg, Amanda Woodward, Dimitri Williams

**Affiliations:** Carnegie Mellon University, Pittsburgh, PA, USA; School of Information Studies, Syracuse University, Syracuse, NY, USA; Michigan State University, East Lansing, MI, USA; DePaul University, Chicago, IL, USA

**Keywords:** older adults, voice assistants, health information seeking, code-switching, speech recognition, identity, cultural relevance, race

## Abstract

Black older adults from lower socioeconomic environments are often neglected in health technology interventions. Voice assistants have a potential to make healthcare more accessible to older adults, yet, little is known about their experiences with this type of health information seeking, especially Black older adults. Through a three-phase exploratory study, we explored health information seeking with 30 Black older adults in lower-income environments to understand how they ask health-related questions, and their perceptions of the Google Home being used for that purpose. Through our analysis, we identified the health information needs and common search topics, and discussed the communication breakdowns and types of repair performed. We contribute an understanding of cultural code-switching that has to be done by these older adults when interacting with voice assistants, and the importance of such phenomenon when designing for historically excluded groups.

## 1 INTRODUCTION

Adults aged 55 and older now make up 29% of the United States population [16], and are increasing annually in their technology ownership and acceptance of technological devices [4]. According to NPR [75], voice assistant devices such as smart speakers are now in 24% of homes across the United States, with 43% of owners reporting using voice assistant devices more often in the last year due to staying at home during the global health pandemic. More specifically, of the older adult households that are equipped with broadband wifi, 34% report using intelligent devices such as smart displays or voice assistants in their home [99]^1^. Thus, the potential for these devices to provide daily support such as companionship and access to information that would aid in self-maintenance of health management is higher than ever for older individuals. There is promise for these devices to be instrumental in making healthcare more accessible. HCI and design research studies examining this potential have asserted that these devices have many benefits for self-maintenance and health information seeking tasks including nutrition advice, tracking water intake, and even managing stress levels [94, 98], but also pose usability barriers for novice users such as older adults learning how to engage with them [59, 93]. In many cases, voice assistants are being designed and developed only with older adult samples who have either access to the Internet or have familiarity with technology. More specifically, groups who are lower-income, who have lower digital literacy or technology proficiency, or who identify with racial identities that have been historically excluded from societal innovation are also left out of consideration of such technologies. As a consequence, we know less about interactions and experiences with voice assistants among subgroups of older adults that may experience additional barriers to technology engagement related to demographic factors of identity outside of age, which have been found to be important factors of interaction [60]. Particularly, assessing the use, perceptions, and experiences of voice assistants for managing health and wellness needs among Black older adults in lower income neighborhoods has great significance and potential impact on future research, yet is relatively absent.

When considering the relevance and usability of voice assistants for Black older adults, there is a need to understand the reception of these devices as health information resources and the perceptions of interactions with these devices. Similar research in the area of cultural reception of speech recognition technologies has begun to examine which languages, accents, and dialects are well received [46, 60], an area which up until this point has primarily been studied in HCI among communities who consider English as their second language (e.g., [35, 52]). This research has engaged with primarily non-White, educated, affluent, or STEM-educated technology users and has observed users’ need to code-switch as being the most prominent theme, which is considered switching between multiple languages, when interacting with smart devices that employ automated voice responses [35, 52]. Even though there has been a lot of research on code-switching with voice-controlled assistants, none of these studies have been conducted with Black populations where there is a particular cultural meaning of code-switching, let alone Black older adults in lower-income environments. Here we reference a form of “cultural code-switching” which encompasses several aspects of behavior, including dialects which mix words that may not be widely recognized as a formal foreign language, but that have lineage in African languages and languages of those who were enslaved in the United States [3, 27, 53]. It is critical that HCI research explore the cultural relevance of this form of code-switching, and the cultural relevance this may have for technology interactions, leading to blatant disparities among racial groups in speech recognition [60]. For certain subgroups of the aging population, this also means additional barriers to digital resources that may impede potential improved health outcomes.

Therefore, in this work we sought to answer:

RQ1: What are the health information needs that Black older adults in lower-income environments would feel comfortable asking online resources?

RQ2: How do they ask health-related questions to online resources such as voice assistants in their homes to fulfill their health information needs?

RQ3: What are their experiences and perceptions as they engage with voice assistants as health information resources?

To this end, we conducted a three-phase exploratory study and engaged in content and linguistics analysis of the types of questions asked and areas of search among 30 Black older adults. In addition to comments of credibility, trust, and resource preference, we found a consistent theme of the code-switching that participants felt was necessary to engage with voice assistants that are not designed for use among those whose dialect may veer from commonly understood proper English. In this paper we identify the health information needs and common search topics, and discuss the communication breakdowns and types of repair performed by this sample of Black older adults, which to date have not been explored among this population in the HCI space. We contribute to the HCI and design space by framing the analysis of a dialect which may better align with the concept of cultural code-switching than as a formal foreign language as recognized by current machine learning or natural language processing research. We discuss implications for design and HCI research, as well as the inclusion of those at the margins in innovative health interventions.

## 2 CULTURAL CODE-SWITCHING

In order to frame our exploration of code-switching in Black older adults’ experiences with voice assistants or other conversational devices, we ground the phenomena of code-switching across ethnic and racial groups as it is studied by anthropologists and linguists. Formally, code-switching is defined as the concept of altering language between two or more native and foreign languages or varying language and is often seen among multilinguals. A term defined by sociolinguists [15], this phenomena has widely been studied as a way to examine how people choose their “linguistic repertoire” [14] and assign certain languages based on what a situation requires [2]. Scholars have likened code-switching to a social strategy that not only helps to translate content in certain social environments but also shows social conformity [72]. Generally speaking, code-switching is a behavioral adjustment that involves shifting one’s appearance, expression, and speech in order to fit into one’s current context and environment.

Research studies examining code-switching among various cultural subgroups look to this phenomena as a way to explore race, ethnicity, and cultural behaviors. Observations of human behavior more broadly suggest that people subtly change their expression all the time based on environment and situational context. Code-switching has different meanings among different minoritized and racial groups. For groups with minoritized and/or racial identities, switching into different accents or languages inadvertently may happen more often or require more mental tasks [17]. In a research study by Carbado and Gulati [17], researchers found that code-switching often takes place in environments where individuals feel there is an “appropriate” behavior or particular norms are expected. In a way, code-switching allows many racial groups to hide in plain sight because they are able to have conversations that have special meaning, significance, and understanding to their cultural communities, building relational histories of language and rhetoric.

Scholars have written of code-switching among Black Americans as a strategy to successfully navigate spaces and environments where they are not just the minority, but may have experienced exclusion, prejudice, and segregation [70]. In studies of workplace culture, Adiah Harvey Wingfield acknowledges how the feeling of needing to code-switch is actually one of social survival, and one that may be done to counter common cultural stereotypes [100]. Here, code-switching may involve engaging in performance that is societally considered more professional or proper in order to sustain mental well-being, economic advancement, and in some circumstances, physical survival [86]. In a larger societal context, code-switching may be a particularly tasking behavior: *“Code-switching is not easy to do and can bring coping fatigue, confusion, missteps and distraction”* [64]. A part of this performance is also the way language is engaged. Scholars have noted that code-switching for many Black Americans also means altering language that may not be recognized as formal dialect, but that stems from African-American Vernacular English (AAVE) or African languages [25, 58, 88].

For a while, scholars debated acknowledging AAVE as a formal dialect or language versus a passing fad of slang only used by younger Black Americans. Scholars such as Rickford [88] and Pullum [84] argue that AAVE is a dialect that follows a systematic set of rules for grammar and pronunciation, mixing words that may not be widely recognized as a formal foreign language, but that have lineage in African dialect and have evolved over the history of Black Americans in the United States. Sociolinguists and language scholars have acknowledged the harm of dismissing the linguistic history of Black Americans as being *“Anti-Black linguistic racism”* [6]. This refers to the “linguistic violence, dehumanization, and marginalization” of Black language and dialect as “lazy” or grammatically incorrect - placing it as integral to the conversation of code-switching. Baker-Bell argues that although considering code-switching that involves AAVE may actually validate this dialect, it also calls for interrogating the cultural hegemony that demands such code-switching in the first place. Considering the legitimacy of the cultural code-switching of AAVE has most commonly happened in research areas of education and classroom inclusion [6, 57], but this interrogation may also be seen as relevant in the technology space. The concept of cultural code-switching provides a model for thinking about behavioral adaptations when engaging with technology through a lens of race and cultural resonance. Our research builds on this foundational understanding of this cultural phenomena to understand how this may influence experiences with technologies that predicate on a cultural standard of English dialect.

## 3 RELATED WORK

To better understand linguistic performance amidst Black older adults’ experiences in using voice technologies for health information seeking, we review literature in the following three areas: 1-perceptions, acceptance, and use of voice assistants among older adults, 2- cultural reception of voice assistants among various racial groups, and 3- language performance with automated speech recognition technologies.

### 3.1 Older Adults and Voice Assistants

Voice assistants provide the potential to support everyday tasks in the home for many consumers [13, 61], and this has become a growing area of interest among researchers focused on supporting health and well-being among older adults with intelligent and smart technologies. The promise of voice assistants as a means of empowering older adults through supporting management of chronic health conditions and illnesses, medication, or even personal health records among has led to scholars exploring the feasibility, usability and ideal interactions associated with these devices [13, 61, 68]. Previous studies have determined that voice assistants are positioned to help older adults who are novice technology access a range of information related to shopping or task organization and engage in leisure activities such as playing music or games [13]. In a study by Martin-Hammond and colleagues [68], researchers found that older adults felt that voice assistants may be useful tools for providing recommendations or alerts, and may also support common interactions with caregivers. Similarly, Koon et al. [61] determined that older adults found voice assistants beneficial for household tasks and inquiries related to daily routines such as asking about the weather. In both studies, perceptions of voice assistants were collected among older adult samples to understand reasons for device adoption and desired scenarios for use. Martin-Hammond and Nallam’s work explicitly identified perceived advantages of these devices in the realm of health and health information seeking, stating that older adults would value being able to reason about health-related symptoms, and gain advice that was personalized to their health status [68, 73].

Among HCI literature scholars have considered older adults’ needs in adopting voice assistants and strategies to best engage the use of these devices for this population [1, 80, 82, 85, 93, 97]. Work by Pradhan et al considered how older adults ontologically categorize voice assistant devices and how designers might move forward in developing these and other devices for the social companionship of older adults [80]. This study found that since older adults tend to anthropomorphise voice assistants, more dynamic interactions may better support what older adults see and feel to mitigate low adoption. In exploring ideal interaction experiences and design specifics, researchers have begun to take a participatory approach by not just exploring use patterns but also collaboratively envisioning future scenarios and configurations to support better experiences [67, 81, 90, 97]. Across many of these studies we see that voice assistants may provide independence, socialization, and information exploration for older users, yet little work has focused specifically on interaction experiences.

Thus far this research area has assumed older adults to be defined as a homogeneous group where researchers have considered age and functional limitation as the primary determinants of health outcomes and usability [92]. Few studies have looked specifically at voice assistant interactions among lower-income older adults to address the digital divide that permeates technology use [73], yet cultural backgrounds and race have not been a part of this conversation^2^. Bickmore et al [12] had a predominantly Black sample of older adults but discussed the need for inclusion of lower economic status without mention of race or cultural background as having a correlation to device use or as impacting access to these technologies. Experiences with conversational technologies among racially minoritized groups who may identify with different language and dialect has yet to be explored, suggesting a need for an in-depth understanding of how demographic characteristics such as cultural background, socioeconomic status, and racial identity may impact the perceptions and reception of voice assistants for health information seeking.

### 3.2 Cultural Reception of Voice Assistants

Prior HCI research has established the importance of making aspects of an interface culturally relevant, such as text, images, colors, and modes of interaction [32, 39, 66, 95]. More recently, we have begun to see this same attention to voice interfaces, with studies like Mendu et al. [71] highlighting the importance of culturally-informed voice assistants to educate Hispanic women about cervical cancer. Disparities and biases (e.g., racial) that can exist in automated speech recognition further reiterate the importance of designing voice assistants that are culturally-informed [60, 91].

Across HCI research we also see that voice assistants’ use and perceptions differ based on different aspects of one’s identity including socioeconomic status, culture and background, and race. Garg and Sengupta discussed race and socioeconomic differences in technology use and non-use among Asian Indians and White Americans in the U.S., and illustrated that these constructs correlate with and ultimately impact reservations parents have about children using voice assistants [36]. Nallam et al also explored the construct of socioeconomic status by examining lower-income older adults’ perceptions of intelligent voice assistants as a way to improve access to these devices [73], suggesting challenges such as cost of maintaining Internet connection as a potential barrier to use.

Thus far, research into the use of voice assistants among Black populations has been primarily situated among younger adults and guided by likelihood of a particular health status. Design and evaluation of various embodied and non-embodied voice agents were proven to be an effective method of informing, motivating, and promoting health behavioral skills in HIV-positive Black young adults [29]. Jack et al designed and evaluated an online preconception conversational agent system and was found to reduce preconception risk in young and educated Black women [48]. Although these and other studies [78] indicate the promise of voice assistants among subsets of the Black community, researchers have acknowledged the cultural barriers that impact experience with these devices. Kim et al. [54] indicated that voice assistants can best serve Black populations by embodying their culture (e.g., giving culturally sensitive health advice) and addressing their privacy and security concerns. Through their preliminary work, Gupta et al. [41] also highlighted that a dialogue voice agent can best serve Black and LatinX patients if they get to interact based on their own initiative, rather than a system that operates as a health educator. Overall, while voice assistants can be helpful among Black populations in their health care management, they are most effective when they are culturally relevant and informed.

Koenecke et al’s analysis of automated speech recognition through transcribed audio clips set a precedence of our understanding of the racial bias that exists in Black American’s use of voice technologies [60]. This study established that many off-the-shelf speech recognition devices exhibit “substantial racial disparities” when transcribing Black voices on audio recordings versus white voices. Koenecke’s work provides foundation for a more in-depth qualitative analysis of the actual experiences of these groups during voice technology interactions, specifically among Black older adults from lower-income environments who have historically experienced additional challenges with technology use and adoption [30].

### 3.3 Considering Language Performance in Voice Assistant Use

Research has highlighted that non-White communities who have lower literacy or digital literacy such as farmers in India are receptive to voice assistants because of the similarity to human-human conversational interactions, despite struggling with voice input [49]. Use of these devices can be challenging due to one’s limited literacy in English, higher comfort in speaking in their vernacular language/dialect which are often not supported by such devices, and/or limited exposure to such devices [36, 89]. In many instances, non-native English users ‘perform’ Standard English with voice assistants for either the benefit of practicing English [26, 35], or to present themselves as upwardly mobile and gain legitimacy as English speakers [51]. In a Western context, children and adults alike are known to often face communication breakdowns while trying to interact with voice assistants, which leads them to shift their way of conversing to be more in line with traditional Standard English and employ various repair strategies to be understood by the device [8], at least in the initial phases of these interactions [37]. These breakdowns are not only because of users’ limited competency of language [8], but also can be attributed to the unintentional errors introduced in their speech while ‘performing’ the language in real and concrete situations [22] as well as the affordance of technology itself [21]. Even older adults are found to be unfamiliar with the workings of voice assistants and the need to use a wake word, oftentimes ascribing limited self-competency to be the reason behind agents’ functional errors [56].

There is a considerable amount of literature that has examined how voice assistants can make the school environment more conducive to learning for children who speak dialects such as AAVE at home and are often expected/forced to code-switch into “standard English” (e.g., [20, 33, 74, 87]). Much of this work however has sought to train AAVE speakers to shift to more standard English [20, 33, 87]. Research by [74], is one of few that acknowledges the value of training conversational technologies to be more inclusive of AAVE by training robots with culturally relevant vocabulary and gestures. Dialects such as AAVE – a language commonly associated with the culture of many Black Americans – are not supported by voice assistants available in the market today and are currently missing from the discussions around designs of voice assistants.

From prior work we know that voice assistants have promise to support older users in the home, yet we do not have knowledge about how this information is sought among Black older adults who come from lower-income environments. Moreover, to the best of our knowledge none of the past work focused on voice assistant use among older adults has specifically looked into needs, preferences, and challenges (e.g. communication breakdowns) related to the use of these devices among Black older Americans. Therefore, this study aims to take the first step towards this direction by understanding how Black older adults in lower income environments perceive and want to use voice assistants to address their health. We expand existing literature to include this subgroup by presenting these perceptions and experiences and the design implications derived from them.

## 4 METHODS

**Figure 1:**
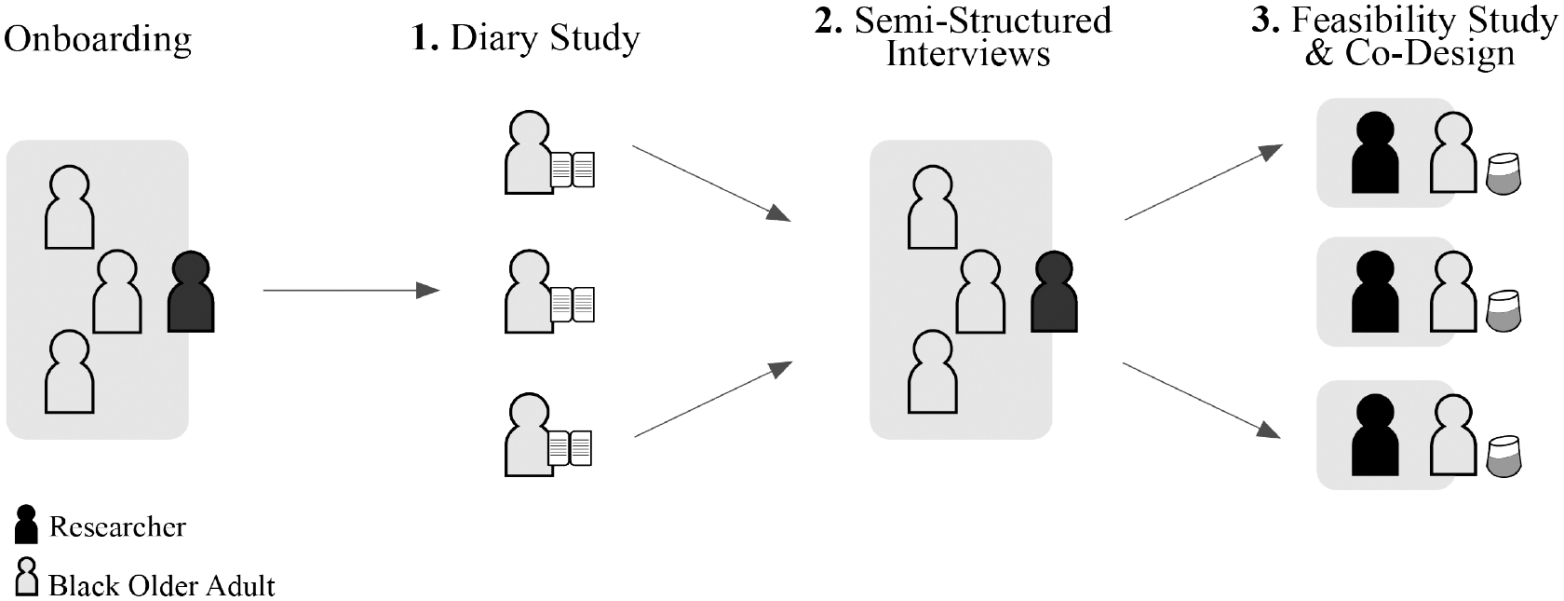
A diagram of our study procedure. Participants were on-boarded in small groups prior to the diary study. The 5-day diary study was followed by follow-up interviews to discuss their diaries and thoughts on online health information resources. This was then followed by a feasibility study where Black older adults communicated with the Google Home and shared feedback on their experiences.

We conducted a three-phase qualitative study to understand how Black older adults from lower-income neighborhoods ask health-related questions to conversational assistants and their experiences with and perceptions of conversational assistants to support health information seeking. This study included a mixed-method, triangulation research approach to validate any findings [19, 62]. Our study combined the following qualitative, exploratory methods in a three-phase study (Please see Figure 1. We discuss in detail in section 4.3):

1. Diary study for real-time elicitation of health-related questions;
2. Semi-structured interviews with low-tech probes of non-conversational and conversational online health information resources to elicit preferences and opinions;
3. Scenario-based feasibility testing with the Google Home and co-design of ideal features.

### 4.1 Recruitment and Community Partners

Participants came from two Midwestern cities that have comparable Black populations both in socioeconomic demographics and in percentage of overall population. Based on an ongoing relationship with local community partners, we recruited Black older adults lower-income neighborhoods from Chicago (n=13) and Detroit (n=21). Initial research activities were held within community settings to provide convenience for participant engagement and to mirror previous HCI research which suggests that situating design engagements within the communities they are intended for can help to promote a level of collectivism in the research itself [43, 45]. In Chicago, participants were recruited via the first author working closely with resident service coordinators at local community centers and residential living facilities that hosted the target population. The first author worked with community coordinators to develop the study and identify potential research participants and attended several community meetings and center-organized events to engage with potential research participants and discuss the ongoing research. Our project description and protocol were then approved by the Community Advisory Board of the Healthier Black Elders Center in Detroit, Michigan, which consisted of Black older adults in our target demographic [42]. Following approval from the university Institutional Review Board, research flyers were posted on the community boards at these centers and shared via a listserv associated with Black elders in a particular metro area. We also used word-of-mouth and snowball recruitment to identify potential participants. This research took place between early March of 2020 and January of 2021.^3^

### 4.2 Participants

In order to participate in this research study, individuals had to meet the following inclusion criteria: (1) be between the ages of 50 and 89 (this was based on the inclusion ages of residents and members of the community centers we partnered with); (2) identify as Black or African-American; (3) reside within certain pre-identified low-income neighborhoods in Chicago or Detroit; (4) be able to read and write to fill out surveys and questionnaires.

We recruited 34 participants at the beginning of our study and collected demographic information. Four participants dropped out due to Wifi connectivity or health issues, thus we analyzed data from the remaining 30 participants (25 women, 5 men). Due to the pandemic, our research protocol was adapted to be conducted virtually through remote Zoom sessions. As such, participants that participated remotely (n=19) were required to have access to a computer that allowed for video conferencing, whereas 11 of our participants reported not owning or having access to a computer. Overall, our sample included elders with varying experiences with technology. Across our sample, 23 participants were retired, 3 were employed part- or full-time, 2 were unemployed, and 2 identified as homemakers. During data collection we collected background experience with both stand-alone and mobile voice assistants. Most participants had never used them prior to this study (n=17 for stand-alone devices, and n=16 for mobile voice assistants), with a small group of these participants not being familiar with what these technologies were (n=3) and (n=2) respectively. Roughly half of our sample had used one or the other at least once (See Table 1). Our sample was heavily skewed towards educated older adults and we note this as a potential limitation of our work.

### 4.3 Procedure

We employed a three-phase study to address our overarching research question of understanding how Black older adults in lower-income neighborhoods ask health related questions of online health information resources (See Figure 1). Prior to the beginning of these three phases, participants were on-boarded either in small groups or individually where all research materials were introduced and initial background questionnaires were administered. Researchers used low-fidelity mockups to introduce websites that may be used for health searches (Google, a health-related website such as WebMD, and a local hospital website) and standalone and mobile voice assistants such as Google Home or Siri, respectively. During this on-boarding, the research team also introduced the paper diary and provided instructions for the 5-day diary study that made up Phase 1. For each of the 5 days, the diary featured an image of the website or device, a brief blurb that explained the resource, and a prompt to list health-related questions they’d ask the resource. Based on previous work [77], a diary study was designed to capture questions that participants may have outside of in-person research sessions which allowed for more convenient collecting of these questions. Capturing health information behaviors in real-time through convenience research methods such as diary studies has been proven effective in previous research efforts [23, 31].

In Phase 2, researchers led participants through group or individual semi-structured interviews about their diaries, as well as their perceptions and attitudes regarding non-conversational online health information seeking resources that were introduced during on-boarding. Participants were guided through conversations about their perceptions and attitudes regarding these resources, and presented with health information seeking scenarios based on questions from participant diaries (see Table 2). Participants were then asked to detail on sticky notes how they might search for the associated health information and to place those sticky notes on the interface mock-up of the resource they would prefer to use. Short discussions followed each scenario about why each participant chose the resource they did and what other ways they might use that resource. Interviews were concluded with researchers asking participants if there were other health-related questions they might search with any of the online-health resources and how they might search for this information.

Phase 3 of the study was held with participants one-on-one^4^. Here, researchers introduced a physical Google Home device and provided participants with a printed handout with a picture and written blurb on features and potential uses, as well as how to wake up the device and give commands. Researchers explained current capabilities and potential uses of the device and prompted the participant to try a few test prompts to get acclimated to giving verbal commands. Participants were asked to test how to wake up and give commands or ask questions to the device using any verbal test prompt that they wished. This was done to also ensure that the Google Home could hear the participant without technical difficulty. Following this, participants were guided through the same series of scenarios from Phase 2 (see Table 2) and instructed to search for relevant health information by asking the Google Home device questions that would help them obtain the information they were interested in. To evaluate the participant’s experience using the Google Home, the researcher then asked participants about whether they would trust answers that came from the device, whether they felt the information was credible, and if they would want to check an additional source to confirm the information. Participants were also asked to state how easy or hard they felt it was to ask health-related questions of the device, and if there was any additional information they wanted to ask the device. The remainder of the Phase 3 sessions included a co-design and brainstorming session to elicit ideal attributes of the Google Home. For purposes of this paper, we analyze and report on the one-on-one sessions of Phase 2 and 3 to understand the questions and prompts given to the Google Home.

### 4.4 Data Analysis and Positionality

Individual and group interviews were audio and in some instances video recorded, transcribed, and checked to ensure accuracy. Search queries were collected from .html files after each participant interacted with the Google Home. We extracted questions and commands from search queries to analyze for themes and frequency counts. Thematic analysis was used to identify patterns based on previous HCI research analyzing similar data [47, 65, 76]. A set of codes were developed by reviewing transcripts and our notes from follow-up sessions and split among the original research team to be coded. We then split interview transcripts to code them to look for patterns in participant experiences and to identify the primary issues described in the interviews. Transcripts were coded until all new and relevant themes had been identified. Background and health demographic data were analyzed for descriptive statistics. Recordings were reviewed against query data to assess communication breakdowns and the types of repairs used. To identify types of strategies our participants used for repairing communication breakdowns we performed a linguistics analysis through semi-open coding, i.e., we began coding participants’ communication repair strategies based on the taxonomy defined by [8] and also identified new strategies inductively if a need arose.

To ensure that we could adequately address any potential biases in examining a theme such as code-switching through content analysis of qualitative data, we engaged a diverse research team with varying levels of education and experience in analysis which served to check assumptions of the data. Data was collected by a Black PhD-educated researcher from a Southern region of the United States. Coding was done by a mixed-race Puerto Rican and Black male graduate student from the Midwest. Data was then further analyzed with a white, middle-class, female researcher who grew up in the Southern U.S. but has lived in the Midwest for most of her adult life and a female researcher who was raised in urban India and identifies as an Asian Indian immigrant to the U.S. and belongs to the middle-class, both of which hold higher education degrees. Our research team was comprised of scholars who had ample experience doing research with older adults, focusing on racial disparities in health services, and understanding categories of technology users based on socioeconomic status, age, and cultural background. All authors were involved in the analysis of data through conversations about themes and interpretations that emerged.

## 5 FINDINGS

We discuss our findings in three sections based on our research questions and the types of communication breakdowns and repairs that emerged during participants’ interactions with the Google Home: Health information wants; Communication breakdowns and repairs; and Feelings of code-switching. As we did not see any difference in-between groups of the two cities across these themes, we report our findings of the overall sample.

### 5.1 Health Information Wants

On average, participants made between 12 and 13 queries each with a minimum of 4 and a maximum of 31. These queries encompassed a wide range of possible uses and sub-categories for the Google Home including music, weather, and small talk, similar to findings from [11]. In terms of health information, participants noted seeing the potential of voice assistants to support access to and understanding of lab work and help with healthcare management tasks such as scheduling appointments and medication reminders. Our analysis found that most consistently, Black older adults in our study made inquiries related to managing an existing chronic illness or condition (searched n=39 times) or medication interaction and dosage information (n=39) (See Table 3 for frequency counts and examples of each category of health information want)^5^.

Participants were also particularly concerned with understanding the target range for vital signs associated with specific conditions and how to stay within those ranges. For example, P31 noted *“What questions would I ask? I will probably want to be specific- uh, a 62-year-old African American woman, that’s obese and pre-diabetic. What’s the numbers I should be looking for.”* Another participant asked Google Home about numbers as well as how to ensure they are reaching those numbers: *“Well, I wanna find out more details about high blood pressure. What are the basic ranges that are good to have, and then also what I can do to lower the blood pressure, and then what types of foods help?”* (P38).

### 5.2 Communication Breakdowns and Repairs

Many of our participants were interacting with Google Home-like devices for the first time. As such, we sought to understand these experiences and perceptions through the feasibility portion of the study. From this, some participants commented on appreciating being able to interact with technology using their voice: *“the good thing about voice assistants is it gets to know your voice, and you can speak it without all that typing and trying to find all the right words. That makes it very easy.”* (P5). However, many participants were skeptical about using Google Home for finding health-related information because they were reluctant to go through the change it asked of them (e.g., the new modality of interaction, adjusting to the absence of display) or were unsure about the accuracy of the information it provided: *“You know how you’re used to doing things the old way. And so I guess what I have to do is get used to change. You know, sometimes it’s hard to change.”* (P19). Another participant remarked, *“I’ll ask, certain things that I’ve heard are true. I would check with the chatbot [Google Home] to make sure it’s okay or it is accurate.”* (P23). Those who were open to the idea of using Google Home for searching health related information felt they would need to learn and/or practice voice-based interactions with technology before they could get comfortable and competent in using the device for this purpose: *“Like I said, again, I’m not used to it. It’s [a] change. I’m 72 years old, I can’t change.. Maybe if I practiced with it, but right now, I’m not used to it” [in reference to using Google Home for health information]* (P4). Another participant, P3, noted:

“I think that you have to learn your device. You have to learn what to ask, the time limit in asking, and what your expectations are, you know. If you talk all willy nilly, then you’re going to get nothing, but if you ask precisely, and in a timely manner, and then probably be able to write things down if they tell you, you know, if you can, I think it would be helpful.”

As a part of our thematic analysis we identified three different communication breakdowns by defining the common ways that Black older adults in our study experienced challenges with using the Google Home during feasibility testing. For each of these, we identified the common approaches to repair that were observed based on work by [8].

#### Communication Breakdown #1: Initiating Interaction with the Google Home.

The first communication breakdown was in how participants woke up Google Home where the formal wake up words like “Hey Google” or “Okay Google” were required. In many instances, participants would directly begin a command or question or just state “Google” before stating a command or question. For example, P39 initiated Google Home with commands such as *“Google, play some music”* or *“Google, what is a healthy blood pressure”* for 13 out of her 14 queries. Some participants attempted the correct syntax to wake up Google Home but used an incorrect greeting word such as *“Hi Google”* or *“Hello Google”*. In many of these failed attempts participants were trying to replicate social norms of human-to-human conversations where it would not be necessary to use a particular phrase to begin speaking or by using a greeting they are most comfortable with or used to using (“Hi” or “Hello” versus “Hey”). Many participants discussed the frustration of even this small step in interacting with the device: *“Well, I think, I need to work on more specific questions and also remembering how to get Google to respond”* (P39). Similarly, P8 commented, *“if you don’t respond to it right [it won’t respond]… you have to respond like, ‘Hey Google’ or ‘Okay Google’, I didn’t do that”*. Common repair strategies among our sample included over-articulation of the phrase or just the individual word ‘Google’, increasing volume, or repetition of the same phrase until the participant asked the researcher how to correctly address the device or re-read the text blurb provided in their research materials. All but two of our participants found this to be a challenge, replicating data from [18, 82, 83] and suggesting difficulty in remembering voice assistant commands.

#### Communication Breakdown #2: Pacing Queries with the Google Home.

The second common communication breakdown involved the Google Home interrupting or cutting off the question or query before the person could complete their ask. In these instances, the Google Home would begin to give responses to only a part of what was being stated, which in many instances would result in an irrelevant answer. For example, P1 experienced Google Home responding to the first half of their question about diabetes prevention *“I’m a borderline diabetic, is there any information on preventions for diabetes?”*. Google Home captured the query as *“I’m a borderline diabetic, is there any information”*, and gave more generalized information on glucose intolerance. Participants commented that they felt the need to be more meticulous in formulating their questions or “asks” of the Google Home, which often caused them to pause and take more time. P5 commented, *“I think that you need to be precise, you know, like I said, you have to, I think before you talk to it, you have to be precise.”* Similarly, P26 details the preparation needed by saying: *“our voice has to be clear. And you’re gonna kinda think out what you’re gonna ask, so it comes out the right way. It’s just like talking to a person so that you can get… You say something, you want them to understand what you’re saying.”* Here we observed both prosodic changes and overarticulation as repair strategies.

#### Communication Breakdown #3: Queries Not Understood by the Google Home.

The final communication breakdown experienced was participants getting a response that Google Home did not understand the question and not providing any further information. For each of these interactions, Google Home would respond with statements like *“Sorry, I don’t know how to help with that. But I’m always learning”* or *“Sorry, I don’t have any information about that”*. In some instances this was due to the content of the older adult’s request (e.g. *“Hey Google, recite the ‘Our Father’ prayer”*), but other times it may have been due to how the question was formulated, both syntactically and semantically. In her attempt to find information pertaining to diabetes, P02 prompted the Google Home with *“Hey Google, how do I find if I’m diabetic”*. When corrected to *“Hey Google, what do I do if I’m diabetic”*, P02 received information such as early signs and symptoms which addressed her original ask. Similarly, P06 wanted to know more about the types of blood pressure medication available, asking, *“I use several high blood pressure medication I can’t think of the name, can you give me some names?”*, to which Google Home replied that it did not know how to help with that. When this response was restated as *“I want you to tell me the blood pressure medications on the market”*, Google Home responded with a list of 8 medications based on the website healthline.com. Here participants most commonly displayed semantic adjustments and modifications as repair strategies.

Many participants felt responsible for Google Home being unable to comprehend their queries and blamed their own technological skills for not being able to effectively communicate with the device: *“I think it’s going to be the same regardless if I, if it’s a voice-response item or typing it in; you still have to find the way to make it make sense for a computer”* (P10). This led them to engage in meticulous restructuring and re-wording of queries in ways they thought Google Home might understand: *“Sometimes I ask Google for something and it will tell me one thing but that is not what I asked them. You know, I have to repeat something, okay you gotta use another way because sometimes it’s how you have to word it in order to get the right answer.”* (P2). Another participant noted: *“Yes, I needed to be more specific in order to get the answers that I wanted, that I need to think about the question”* (P30).

### 5.3 “Everybody Doesn’t Use the Same Language”

Another observation was that of question abandonment when participants realized that the Google Home would not be able to understand them. Here, Black older adults in our study would ask a question and upon feeling that they experienced one of the aforementioned communication breakdowns would actually abandon the question altogether and choose to ask something entirely different. This differs from instances where participants changed the syntax of the intended question or their speech rate while keeping the content the same. Participants commented here that Google Home either did not know the response or, more prominently, that Google Home and other voice assistants were unable to understand them, either because of their voice, or the way they talked,

“My voice just doesn’t register clearly with it. I have one of those Echo Dots, like won’t say your name. And she understands me a little bit better, but even then… And I use her a lot, like when I’m cooking, and even then I’ll get, ‘Sorry, I didn’t get that,’ or she doesn’t know the answer”

(P29).

Whether engaging in a formal communication repair or abandoning a question altogether, subsequent attempts observed highlighted a unique pattern of communication repair that many Black older adults in our study described as the need to “code-switch” to be better understood by this and other conversational devices. Our participants felt that Google Home struggles to understand their communication style (e.g., diction or accent) and language (e.g., dialect) specifically due to the device being based on Standard English: *“You do have to change your words. Yes. You do have to change your diction and yes, you have to use… It cannot be an exotic name or a name that’s out of the Caucasian round. …You have to be very clear with the English language. No ebonic.”* (P31). Another participant, P39, was curious if it responds to “non-standard English dialects relative [to] different ethnic groups”, and noted, *“I don’t think they would respond too well to slang or any idiomatic… Is it idiomatic or idioms of sub-cultures that we have in our country, so you have to be…You must speak standard English to be understood by it. I would imagine.”*

Participants also noted their perception of a language disconnect with the Google Home where they perceived vocabulary to be the differentiating factor. For example, P26 noted: *“And I keep going back to that saying it so like you’re talking to a person so they can understand you, but everybody doesn’t use the same language, or have the same vocabulary, everybody is different.”* Another participant commented on having to change their expression (tone of voice) and verbiage in a way that is comparable to the code-switching they regularly engage in based on social context and environment:

“I think that there could be a tendency to speak a little more clearer than your actual conversational dialect… Or you might change words, it’s kind of like a code-switching… We do it all the time. We do it all the time now, depending on who you are talking to”

(P11).

In many instances participants mentioned having to switch from speaking in informal contractions, some commonly used such as “gonna” or “wanna”, and some more closely tied to AAVE such as “fixin”. Some participants mentioned assuming ahead of time that they would need to speak closer to Standard Engligh, and therefore taking their time in interacting with the Google Home for the first time. For other participants they mentioned this as speaking in a particular tone of voice that showed less inflection in their voice. Having to code-switch made the process of interacting with the device cognitively-burdensome and time-consuming for the participants, which in turn defeated the purpose, i.e., accessible and prompt interaction, of using voice as the modality of interaction for participants:

“It didn’t feel comfortable, because I had to think exactly what question I was going to ask, what format I was going to ask. I would have to sit down and write some questions down before I would- You know, I couldn’t do it spur of the moment, because then I would have to have some type of way to write down my responses”

(P36).

Another noted, *“No, I’m saying, I want the answer right then. The fact that I have to figure out how to ask the question is kind of …defeating my purpose.”* (P32). Some participants questioned if this would limit the ways the device is used by different cultural subgroups, suggesting potential barriers in voice assistants not understanding those not speaking Standard English,

“I don’t know, that’s what I’m thinking, I’m just wondering, if it would, or is it compatible to pick up all of those little, as they say, little nuances that people have in their voices.. Capture everybody’s voice, even if it’s different, or their language, even if it’s different, or some people have slight accents, some people speak with slurs, you know different things like that, and, is it gonna be able to capture all of that?”

(P26).

P29 also noted that from their experience this would limit the usefulness of the device for other racially minoritized users, *“I can only imagine, I don’t think I have much of an accent, but people tell me it can tell I’m from the Midwest. But my son-in-law has a heavy Spanish accent, so I can’t imagine how he must struggle sometimes trying to use a voice assistant. ‘Cause I have a hard time understanding him.”*

## 6 DISCUSSION

This study explored the use of voice assistants for health information seeking among 30 Black older adults in lower-income areas with varying experience with conversational technologies. Our larger research goal was to understand how these older adults asked health-related questions to inform how we might extend the use of off-the-shelf resources to support health-information seeking among communities that are most often impacted by health disparities. Focusing on Black older adults in particular is important due to the barriers that impede healthcare access such as cost of in-person healthcare engagements [34] and the stigma and structural racism oftentimes experienced by the Black community in medical settings [38, 50]. Although this potential is greatly supported by previous research with other samples of older adults, there is question as to whether these devices will support those who often experience lower technology proficiency and familiarity, and who may feel that such technology is not designed for them, further perpetuating these inequities.

Our analysis explored both the content of these questions and a linguistics analysis of speech interactions with the Google Home. Data triangulation allowed us to capture participant questions in real-time and then go more in-depth with participants to discuss those questions [19, 62], providing a more clear picture of the types of questions Black older adults desired to ask and the natural ways they would pose those questions to online devices. Our findings highlight that while some participants felt there may be value in using the Google Home as an in-home health resource, others felt there would be a notable challenge of not being understood by these devices. While some of the more general search queries from our study such as “diet and nutrition” and “general health and fitness tips” echoed previous explorations of older adults information wants [13, 61, 68], our findings were unique in the ways that Black older adults searched for health information specifically related to their identity (e.g. how many people of color are infected with the coronavirus). This was also seen in participants’ searches for “acceptable range of symptoms”- Black older adults in our sample sought to understand how their current vitals might compare to acceptable ranges by also contextualizing their race and gender stating their belief that these ranges are often different for Black Americans. As these particular searches often resulted in Google Home responses of *“Sorry, I don’t understand”*, there is a gap in culturally-specific information that might support groups like Black older adults becoming long-term adopters of voice assistants. This suggests an opportunity for voice assistant devices to consider search algorithms that are more culturally tailored to users.

Aligning with and extending previous work [55, 56], we found that many older adults in our sample struggled with the mechanics of initiating interaction and wording questions in a way that would support relevant responses. Our study contributes to existing literature by examining the cultural component of voice assistant experiences among Black older adults specifically, with Black elders in our study drawing parallels between navigating speech with conversational technologies and cultural code-switching done in their everyday lives. This provides insight into the ways historically marginalized populations of users perceive the AI technologies that are now pervasive in domains such as healthcare.

### 6.1 Perpetuating Inequities by Ignoring Cultural Identity

At the beginning of our study and prior to actual interactions with the Google Home, many of our participants expressed their assumptions that the Google Home would not understand them and that voice-controlled technologies were not made for them. For many of them, these assumptions were validated by facing communication breakdowns during interactions with the device during data collection. Oftentimes aging research (and older adults themselves) defer to age-related changes as the reason behind older adults’ challenges with technology [7, 63] and as fueling their lack of desire to learn newer systems. Prior research suggests that usability challenges like not remembering how to activate Google Home or communication breakdowns such as misinterpreted commands might be attributed to older adults’ lack of familiarity with newer devices such as voice assistants [18, 69, 82, 83]. However, our data suggests that cultural background is actually more at play as a reason behind breakdowns many of our participants experienced. Here, we actually see the construct of age provide a *positive* framing which contributes context to this phenomena due to many Black elders sharing that they have had to perform cultural code-switching for decades and thus can better articulate the usability challenges and barriers presented by voice technologies that now require them to code-switch. As Pennycook [79] states, individuals often “perform” language to pass as locals in the context that they are in. Black elders have had to engage in this “performance” and behavioral change in human-to-human conversations for so long that participants in our sample reported that they anticipate, and ultimately experience, such interactions with voice assistants. Voice interaction as a modality is supposed to be more accessible and afford quicker experiences, yet for Black elders like our participants, the requirement of ‘linguistic performance’ when interacting with voice assistants negates potential benefits. We observed that many of our participants often abandoned questions when the voice assistant failed to understand them or because they found code-switching to be mentally demanding. As a consequence, we suspect that such experiences may lead to historically marginalized groups whose natural dialect differs from what is defined as Standard English to not use the device over the long term, specifically for health information seeking purposes.

Prior work has shown the benefit of conversational devices such as Alexa or Google Home in non-native English speakers practicing English [26, 35, 51], although for some groups, such as urban Indians who often code-mix while engaging in human-to-human conversations, using voice assistants present unique struggles [35]. In our study, we found that the design of voice assistants has yet to consider AAVE as a dialect and part of a culture that may influence users’ interaction experiences. Ignoring varied styles of interaction and dialects of Black American adults and not examining whether voice interaction with these devices requires certain groups to code-switch across varieties of English [2] may prove to be harmful in the larger scope of inclusion in HCI. Research suggests that for many Black Americans, code-switching is a form of survival, acclimation, and navigating spaces that have historically been unwelcoming and laden with racial tropes and stereotypes [86, 100]. In considering technology interactions, ignoring dialects such as AAVE in speech recognition reinforces societal cultural exclusions. In this way, the design of voice assistants further marginalizes a community that has been traditionally ignored and who assume that these technologies are not intended for them. For this reason, while previous research has identified challenges of voice assistant technologies’ misinterpretations among older adults in general [55, 56], there is a need to examine this phenomenon of cultural code-switching as a part of the cultural experience for Black Americans and a factor that influences the acceptance of these technologies among users that already feel they are not considered the intended user. Black older adults’ perceptions of code-switching as an experience with voice technologies should not be confused or dismissed as lower technical aptitude or proficiency. Instead, anecdotes of voice-enabled or AI-based technologies such as automated translators or autocorrect misinterpreting individuals with accents or dialects that vary from what is considered standard English should be examined as research data points. These considerations serve to build systems that are more inclusive and more widely accepted.

### 6.2 What Does this Mean for Design?

In addition to addressing our research questions, we identify several recommendations that emerged from our findings and serve to support voice technologies being more inclusive of marginalized voices and dialects. We see these as not just recommendations for the design of voice technologies, but considerations that may be more generalizable to technology and systems that may currently overlook groups like Black older adults from lower-income communities and to the HCI research approaches that supports the design of these devices. Currently in the United States, “Standard English” is considered the “correct form” of language, otherizing any language that sits outside of this designation. Because of this, our participants felt that there is a standard or norm for voice interactions and they fall into the “non-standard”. This in turn positions language and speech recognition in technology as enforcing its own power dynamic, with the current design of voice assistants being culturally hegemonic and demanding Black Americans and others to code-switch [5], rather than adapting design to the needs and preferences of users. Ruha Benjamin in *White Supremacy and Artificial Intelligence* [10] notes that *“Whoever defines the standard expression exercises power over everyone else, who is forced to fit in or else risk getting pushed out.”* By defining the standard expression of language, speech recognition perpetuates its own form of erasure, control, and colonization. Thus, findings from this study recommends ***recognizing dialects such as AAVE in the design of voice technologies***, extending Koenecke et al’s call to audit machine learning systems to ensure inclusivity [60]. Recognizing dialects like AAVE considers language as more of a melting pot where constructs are borrowed and interchanged, representing the interweaving of American cultures.

Our analysis of the ways Black elders have had to adapt to interact with voice technologies suggests ***shifting the ways we consider older adults and racially minoritized groups as being deficit-based users of these technologies***. There is a larger challenge with how we see older adults in the scope of health tech, oftentimes as a problem to be solved. Future research may be able to follow an assets-based approach [101] to investigate the strengths that come from prior life experience. Among our participants, for example, experience and knowledge of cultural code-switching for decades became the very foundation of how they engaged with the Google Home, even if it was challenging to figure out how to implement that approach. Therefore, we see merit in including voices and dialect of Black elders in the design process itself to ensure different aspects of their intersectional identities are considered in the design of the voice assistants [46]. Scholars and industry professionals alike have begun to see merit in the notion of *“nothing about us without us”*, and call for racial diversity among design and research teams [46]. What we have yet to see is this push focusing on the presence of older users of technology, particularly historically marginalized groups of older adults, in the design and development of emerging technologies.

There may also be merit in ***incorporating Black older adults in the design of the conversational dynamic itself such that it feels more natural to non-technological conversation***. The exploration of conversational dynamics in voice assistants, particularly in the sector of healthcare and well-being as [24] suggests is an area where *“conversation may be appropriate, if not essential”*. Many of our participants commented on how interactions with the Google Home did not feel like natural conversations but instead they had to be meticulous and rehearsed in the way they gave commands and questions to the Google Home. Thus, future work may explore more naturalistic ways of communication that may be more comfortable for communities of color. Currently, people are able to change the voices of the assistant to be a familiar voice or media personality such as Issa Rae, but the actual interaction style or dynamic is far from resembling human-to-human conversation – so much so that scholars have suggested that voice assistants agents can only be inspired by human-human conversation but do not need to mimic it [24]. We do note, however, that having differences between human and machines is important for establishing appropriate boundaries, otherwise, for example, people can perceive it as creepy or might disclose information that could be exploited. Therefore, designers would then also have to critically examine ethical implications of human-like conversations with voice assistants.

Lastly, to address technology inclusion more broadly, there is a need for designers of voice assistants to ***engage in the long-term process of cultural and linguistic divesting of colonial power*** [96]. This requires the field of HCI to reflect on the narrative of standards and norms that are implicit in technology but are also inherent in society. It also requires us to find ways to empower ‘natives’ to deconstruct and reconstruct sociotechnical systems to adhere to their values, culture, linguistic preferences, and belief systems. Benjamin calls for “re-writ[ing] the dominant cultural codes rather than simply code-switching” [9], suggesting that we in the tech industry rethink the system architectures, infrastructures and interaction modalities that do not consider diverse cultures. This may consist of looking to those elements considered to be cultural differences which are often otherized, as positive contributions to our technological fabric, similar to Grimes-Parker’s discussion of acknowledging and honoring cultural heritage in ‘Celebratory Technology’ [40]. To this end, Baker-Bell’s ‘I Can Switch My Language but I Can’t Switch My Skin’ highlights several Black cultural modes of discourse that can be considered in the ways we expand semantics in language recognition [5] such as *‘semantic inversion’* (when a word takes on a reverse or inverse meaning) or *‘signifyin’* (double meaning used to provide playful commentary). In rewriting the ways these systems interact with all users, we better consider historically marginalized users. As Benjamin states, “whereas code-switching is about fitting in and ‘leaning in’ to play a game created by others, perhaps what we need more of is to stretch out the arenas in which we live and work to become more inclusive and just.” [10]. In this way, HCI can also contribute to rewriting social norms.

### 6.3 Authors’ Reflection on Conducting this Research

The absence of this nexus of age, income, and race in voice technology research requires some attention on it’s own. While there are empirical contributions that emerge from this study, we find it also pertinent to reflect on our experience conducting such work as a way to encourage other researchers to conduct research in this area. Throughout HCI, there is a perception of barriers in engaging certain populations in research whether due to recruitment, attrition, lack of interest, or cultural disconnects that would prohibit a researcher from being received in a community. Too often we ignore how our position and identity(ies) in relation to the participants, our colleagues, the data, and within the academy influences the research enterprise. We have seen however how leaving these groups to just be considered unreachable in our research also impacts these communities being reflected in the technologies we use, and in some instances further marginalized in society [44]. While it is important to remember that no racial group is a monolith, we must also consider how we approach and discuss topics in HCI related to groups that may find themselves comfortable divulging with researchers who mirror their identity in some way.

The authors acknowledge that the framing of this data may not have emerged if the data collection team did not share cultural identity and ethnicity with the participant sample. Code-switching within the Black community is often something that is understood as a part of the cultural fabric of a racial group who has been relegated to positions of marginalization throughout history. Participants may have felt a level of comfort in dealing with our research team due to racial identity but also indicated feelings that dialects like AAVE and cultural code-switching are notions that are just understood. Previous literature has suggested the value of relationship building and establishing trust with communities. Here, the use of community partners and a participant registry and community advisory board were instrumental in getting participation of this population.

Reflecting on our research approach also requires delving into difficult conversations around racial and social injustice that many researchers may see as outside their purview and are hesitant to navigate. This brings attention to the diversity (or lack thereof) among those doing this area of research and the ways that injustices in larger social structures are mirrored within academia, industry, etc. and enable inequities to continue without being challenged.

### 6.4 Barriers of Digital Access

Racial minority older adults have historically had lower rates of using the Internet due to barriers of cost and access, despite their interest in using the Internet [28, 30]. As there is a current trend of exclusion of this population in more innovative areas of HCI research [44], we focused this study on understanding the experiences and any additional challenges that Black older adults from lower-income environment may face when using voice assistants for health information seeking. Reflecting on our findings and research protocol also highlights that our current research methods for remote studies are limited and face the same barriers that have been commonly associated with the technological divide. Our initial protocol included going into community centers to conduct this study such that those who may not regularly have access to technology might participate and provide insight of their experiences and feedback on the feasibility of these devices as resources for health and well-being. However, conducting some of the research sessions remotely during the pandemic meant that we could only include those who had access to internet and videoconferencing software. Access and cost continue to be a challenge to such technologies being integrated as long-term resources in lower-income communities, and also impact the inclusivity of our research methods. Future work might examine more innovative ways to support access to remote studies for lower-income communities.

However, findings from our study also highlights how other aspects of one’s inter-sectional identity (e.g., race, language spoken) greatly impact technology adoption and use while also potentially exacerbating the digital divide. We see digital exclusion by ignoring cultural identities of people, in our case Black elders from low-income backgrounds, as a new form of social deprivation, aggravated by and contributing to existing lines of inequality and poverty, and as the world gets ‘smarter’, the divide gets wider. Thus, one of the values of this work is considering the needs of historically marginalized population of Black elders in the design of future voice technologies.

## 7 CONCLUSION

The purpose of this study was to examine Black older adults’ perceptions and experiences with voice assistants, particularly how they ask health-related questions and what information they are interested in searching. This work has significance to HCI as this is a group who are often most impacted by health disparities, yet the least represented in research. We observed that Black elders made inquiries ranging from managing chronic health conditions and medication dosage to home remedies and diet and nutrition. From our analysis we found that elders face several communication breakdowns as they attempt to interact with the device. But, more importantly, they felt the need to code-switch when dealing with voice assistants similar to the cultural code-switching done in a larger societal context. In the end, we discuss the importance and ways of including historically excluded groups in designing emerging technologies.

## Figures and Tables

**Table 1:** Participant Demographics & Experience with Voice Assistants. (P01-P11 were Chicago residents, P19-P43 were Detroit residents)

ID (Gender)	Age	Employment Status	Education	Stand-alone voice assistant device (e.g., Alexa, Google Home, etc.)	Mobile voice assistants (e.g., Siri, Google Assistant, etc.)
P01 (F)	72	Retired	Some college	Not used	Not used
P02 (F)	74	Retired	Vocational	Not used	Not used
P03 (F)	67	Retired	Some college	Not used	Used occasionally
P04 (F)	73	Retired	Vocational	Not sure what it is	Not used
P05 (F)	76	Retired	Bachelor’s degree	Not sure what it is	Not sure what it is
P06 (M)	71	Retired	Master’s degree	Used occasionally	Used occasionally
P07 (F)	65	Homemaker	GED	Not used	Not used
P08 (M)	75	Retired	Some college	Used frequently	Used frequently
P09 (F)	71	Homemaker	Vocational	Not used	Not used
P10 (F)	66	Unemployed	Bachelor’s degree	Used occasionally	Used occasionally
P11 (F)	71	Retired	Bachelor’s degree	Not used	Used occasionally
P19 (F)	75	Retired	Some college	Not used	Not used
P22 (F)	74	Retired	Master’s degree	Used frequently	Used frequently
P23 (F)	65	Retired	Bachelor’s degree	Used once	Used occasionally
P24 (F)	66	Part time	Master’s degree	Used frequently	Used occasionally
P26 (F)	70	Retired	Some college	Used frequently	Not used
P27 (F)	65	Retired	Bachelor’s degree	Used frequently	Used frequently
P28 (M)	79	Retired	Master’s degree	Not used	Not used
P29 (F)	67	Full-time	Master’s degree	Used frequently	Used frequently
P30 (F)	72	Retired	Some college	Not used	Not used
P31 (F)	63	Full-time	Bachelor’s degree	Used once	Used once
P32 (F)	72	Retired	Master’s degree	Not used	Not used
P34 (F)	71	Retired	Master’s degree	Used occasionally	Used occasionally
P36 (F)	72	Retired	Bachelor’s degree	Used occasionally	Not used
P37 (F)	78	Retired	Some college	Not used	Not used
P38 (F)	60	Unemployed	Some college	Used once	Used occasionally
P39 (M)	67	Retired	Master’s degree	Not used	Not used
P40 (M)	84	Retired	Bachelor’s degree	Not sure what it is	Not sure what it is
P42 (F)	76	Retired	Some college	Not used	Used occasionally
P43 (F)	78	Retired	Bachelor’s degree	Not used	Not used

**Table 2:** Search scenarios provided to participants.

	Search Scenarios
1.	Imagine you want to find out information about a new medication the doctor mentioned. What questions would you ask the Google Home? Please ask them now.
2.	Imagine you are searching for symptoms of high blood pressure. What questions would you ask the Google Home? Please ask them now.
3.	Imagine you are interested in searching for information on diabetes to understand how you might change your diet. What questions would you ask the Google Home? Please ask them now.
4.	What are other areas of your personal health that you want to search for?

**Table 3:** Health information wants and count.

Health Information Want	Example	Count
Information about managing an existing chronic illness or condition	*“What precautions can I take to lower my blood pressure?”*	39
Information about medication dosage, interactions, or side effects	*“Could you help me about a medication Lasix?”*	39
Understanding or detecting chronic illness including definitions and symptoms	*“Could you tell me more about bronchitis?”*	35
Diet and nutrition information	*“How much sugar do I need?”*	30
Acceptable range of vital signs	*“What number is safe for a pre-diabetic?”*	19
COVID or vaccine information	*“How many people are infected with the coronavirus and how many of them are people of color?”*	15
General health and fitness tips	*“I just stubbed my toe and it really hurts what should I do?”*	13
Disease and illness prevention	*“What can I do to prevent myself from catching the flu?”*	5
Home remedies	*“Can you tell me whether you can drink eucalyptus oil internally?”*	5
**Total**		200

## CCS CONCEPTS

• Human-centered computing → HCI design and evaluation methods; User studies; Empirical studies in accessibility; • Social and professional topics → User characteristics; Race and ethnicity; Cultural characteristics.

## Footnotes

1 While we recognize these devices are also referred to as voice assistants, intelligent assistants, conversational agents, or other, we use the term ‘voice assistants’ for simplicity throughout this paper with no particular distinction between these terms.

2 We do note that not all research studies done with lower-income older adults report racial demographic data.

3 Research engagements were paused from March to October of 2020 due to the global health pandemic which prohibited entering senior centers or facilities.

4 Some sessions were held in person and some were held over Zoom due to the timing of the global pandemic which restricted in-person research activities. Researchers tested the ability of Google Home to pick up voices over Zoom with clarity and precision with the Community Advisory Board prior to engaging with participants

5 The common search for coronavirus and vaccine information may have been influenced by the timing of this study since testing took place between October 2019 and March 2020 and then resumed in October 2020 until January 2021.
